# Does endometriosis inflict harm on embryos? A systematic review of embryo morphokinetics analysed by time lapse monitoring in women with endometriosis

**DOI:** 10.1007/s00404-023-07293-1

**Published:** 2023-12-08

**Authors:** Diana Cupino-Arcinue, Beata Seeber, Markus Montag, Bettina Toth

**Affiliations:** 1 Department of Gynecologic Endocrinology and Reproductive Medicine, Medical University of Innsbruck, Anichstrasse 35, 6020 Innsbruck, Austria; 2 Ilabcomm GmbH, St. Augustin, Germany; 3 Present Address: Department of Obstetrics and Gynecology, Paulino J. Garcia Memorial and Research Medical Center, Nueva Ecija, Cabanatuan City, Philippines

**Keywords:** Endometriosis, Time lapse monitoring, Embryo morphokinetics, In vitro fertilization (IVF)

## Abstract

Endometriosis has been shown to be associated with unfavorable development and maturation of oocytes, as well as aberrancies in embryonal development, including arrest after fertilization, following in vitro fertilization (IVF). Time-lapse monitoring (TLM) enables continuous and non-invasive monitoring of embryo morphokinetics during the IVF process and might be useful in the assessment of embryos from women with endometriosis. In this review, five eligible studies were evaluated to determine if embryo morphokinetics assessed under TLM differ in patients with endometriosis and subsequently predict blastocyst quality, implantation and success of pregnancy. The studies showed overall inferior morphokinetic parameters of embryos from endometriosis patients when compared to controls, independent of the severity of endometriosis. Embryos with optimal early morphokinetic parameters (t2, s2, t5, tSB, tEB) and late developmental events (compaction, morulation, and blastulation) had better implantation rates than those who had suboptimal ranges. However, due to few studies available with mostly retrospective data, the validity of these findings and their generalizability for clinical practice needs to be further assessed. Prospective studies with larger sample sizes are needed to determine whether using TLM for embryo selection in endometriosis improves pregnancy and live birth outcomes.

**Supplementary Information:**

The online version contains supplementary material available at 10.1007/s00404-023-07293-1.

## Introduction

Endometriosis is a debilitating estrogen-dependent inflammatory disease, characterized by the presence of benign functional endometrial glands or stroma outside the uterine cavity [1]. It affects about 6–10% of reproductive-aged women, often causing both chronic pain and infertility [2]. Approximately, 25–40% of infertile women and roughly 25% of patients undergoing in vitro fertilization (IVF) treatment are diagnosed with endometriosis [1].

Endometriosis may have detrimental effects on oocyte quality, as well as potentially inducing aberrancies in embryo development [3]. Numerous studies have shown the unfavorable effects of endometriosis on the normal follicular physiology, including increased apoptosis and dysregulation of molecular pathways involved in development and growth of granulosa cells [3]. Endometriosis is also implicated in altering the microenvironment of the maturing oocyte, observed as an increase in oxidative stress from chronic inflammation [4]. Furthermore, spindle abnormalities in oocytes from patients with endometriosis have been reported. Hence, embryonic development appears to be negatively affected, as confirmed by in vitro studies showing a higher likelihood of developmental arrest in embryos from patients with endometriosis [5].

Historically, morphological grading of embryos at pre-defined but static intervals has been used to assess embryo development, but only provides a limited snapshot of a dynamic process. In contrast, time-lapse monitoring (TLM) continually analyzes embryo development through serial image acquisition every 10–15 min, without disturbing the culture conditions [6]. It provides a unique insight into the subtle impairments of embryonic development, that otherwise would go unnoticed [7]. Several studies have evaluated the impact of TLM for embryo selection as an algorithm-based objective method. Different parameters in embryo development have been found to be significant predictors of implantation and live birth potential: time to division from two to three cells, between division from three to four cells and division to five cells, blastocyst expansion, as well as multinucleation at the two-cell stage [8, 9]. TLM also offers the advantage of stable culture conditions avoiding unnecessary exposure to un-physiologic conditions such as oxygen, pH and temperature changes as well as stress, which are critical to guarantee embryo viability [8]. Therefore, it facilitates the workflow in the IVF laboratory and is an important instrument for non-invasive embryo selection [10].

The quantitative assessment of cell-cycle parameters in embryos derived from endometriosis-affected oocytes may provide further insight into the impact of the disease on embryo quality and development. Hence, our objective was to perform a systematic review to determine if embryo morphokinetics assessed with TLM are suitable for the prediction of blastocyst quality, rate of implantation and pregnancy success in women with endometriosis.

## Methods

### Data sources

We followed the PRISMA Guidelines for systematic reviews. The research was conducted using the following electronic databases: MEDLINE, EMBASE and Cochrane Library. The studies were identified with the use of a mesh combination of the following keywords: *“endometriosis”, “IVF”, “embryo”, “time lapse imaging/monitoring”, “embryo morphokinetics and “embryo quality”,* from the inception of each database to July 2022. The authors independently screened titles and abstracts of studies obtained in the search. All types of studies were selected and each potentially relevant study was obtained in full text and assessed for inclusion. Proceedings of scientific meetings and abstracts were not considered.

### Study selection

All articles describing TLM applied to endometriosis patients during assisted reproductive technique (ART) were considered for review. Only original papers in English were included. The specific inclusion criteria were as follows: cohort, case control studies and observational studies (retrospective or prospective), women undergoing IVF/intracytoplasmic sperm injection (ICSI), study group consisted of women with endometriosis diagnosed by laparoscopy or ultrasound, control group with tubal or male factor infertility, unexplained infertility or mixed etiology infertility and the embryo assessed morphologically using TLM. The exclusion criteria included non-English manuscripts, studies without a control group, conference abstracts, personal communication or systematic review, inclusion of patients with uterine anomalies, polycystic ovary syndrome (PCOS) and premature ovarian insufficiency, or conditions believed to negatively affect oocyte quality.

### Risk of bias assessment

Two authors (DA and BS) assessed the risk of bias in the included studies using the Methodological Index for Non-Randomized Studies (MINORS) [11]. Seven domains associated with risk of bias were evaluated in each of the studies included, namely: (1) Aim (i.e. clearly stated objective), (2) Subjects (i.e. inclusion of consecutive patients and response rate), (3) Data (i.e. prospective collection of data or data collected according to a protocol established before the beginning of the study), (4) Blinding (i.e. unbiased assessment of study endpoints, including blind evaluation of endpoints), (5) Time (i.e. suitable follow-up time), (6) Loss (i.e. loss to follow-up) and 7) Size (i.e. calculation of the study size). Review authors´ judgments were classified as “low risk” (reported in the study), “high risk” (not reported in the study) or “unclear risk of bias” (reported in the study but inadequate).

### Morphokinetic evaluation

Previous work by Cetinkaya et al. and Ciray et al. described which key morphokinetic evaluation of embryo development can be used to determine embryo quality, as summarized in Supplemental Figure. All five studies used the proposed guidelines on the nomenclature of dynamic human embryo monitoring, established by a timelapse user group [12–14].

Cetinkaya et al. suggested an improvement in the morphological assessment of embryos by using relative kinetic expressions [12]. Previously Dal Canto et al. observed significant differences in time in the development from the five- to eight-cell stage and from the four- to eight-cell stage for embryos developing to blastocyst stage compared to embryos arresting after the eight-cell stage [15]. CS2–8, CS4–8, and CS2–4 determine the synchronicity of cell cycles. For example, CS2–8 exhibits the time the embryos spent at two-cell stage and four-cell stage compared to the time from the two- to eight-cell stage. A ratio close to 1 for CS2–8 reflects high synchronicity in the cell cycle regulation among sister blastomeres, signifying an ideal embryonic development. Additionally, the ratios CS2–4 and CS4–8 help assess the synchronicity in the second and third embryonic cell cycle, respectively. Optimally synchronized cell cycle regulation among sister blastomeres will have a ratio close to 0 for both CS2–4 and CS4–8, demonstrating a healthy development of all blastomeres in the embryo up to the eight-cell stage [12, 13].

## Results

**Fig. 1:**
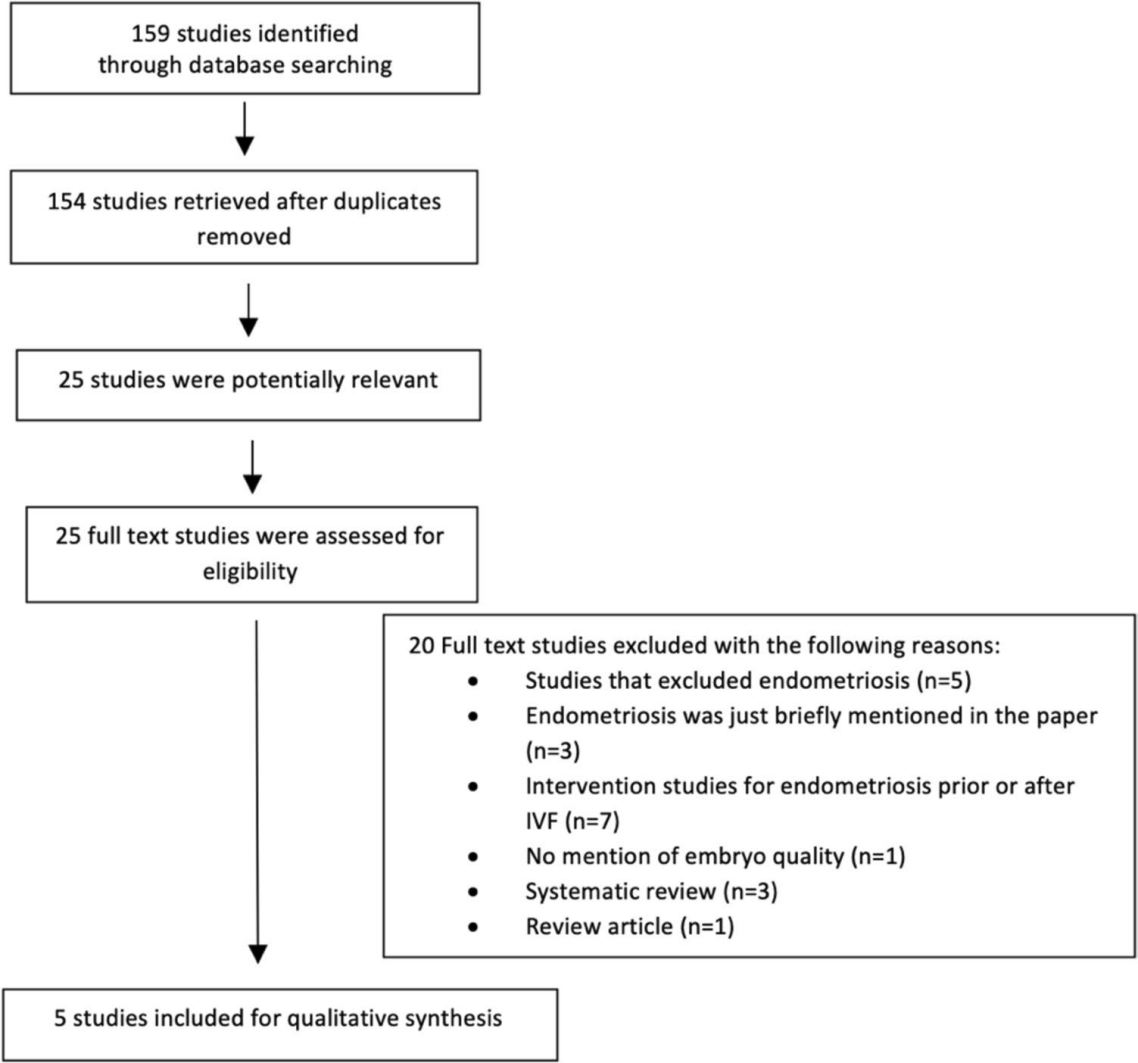
Flow diagram summarizing the systematic review search

**Fig. 2:**
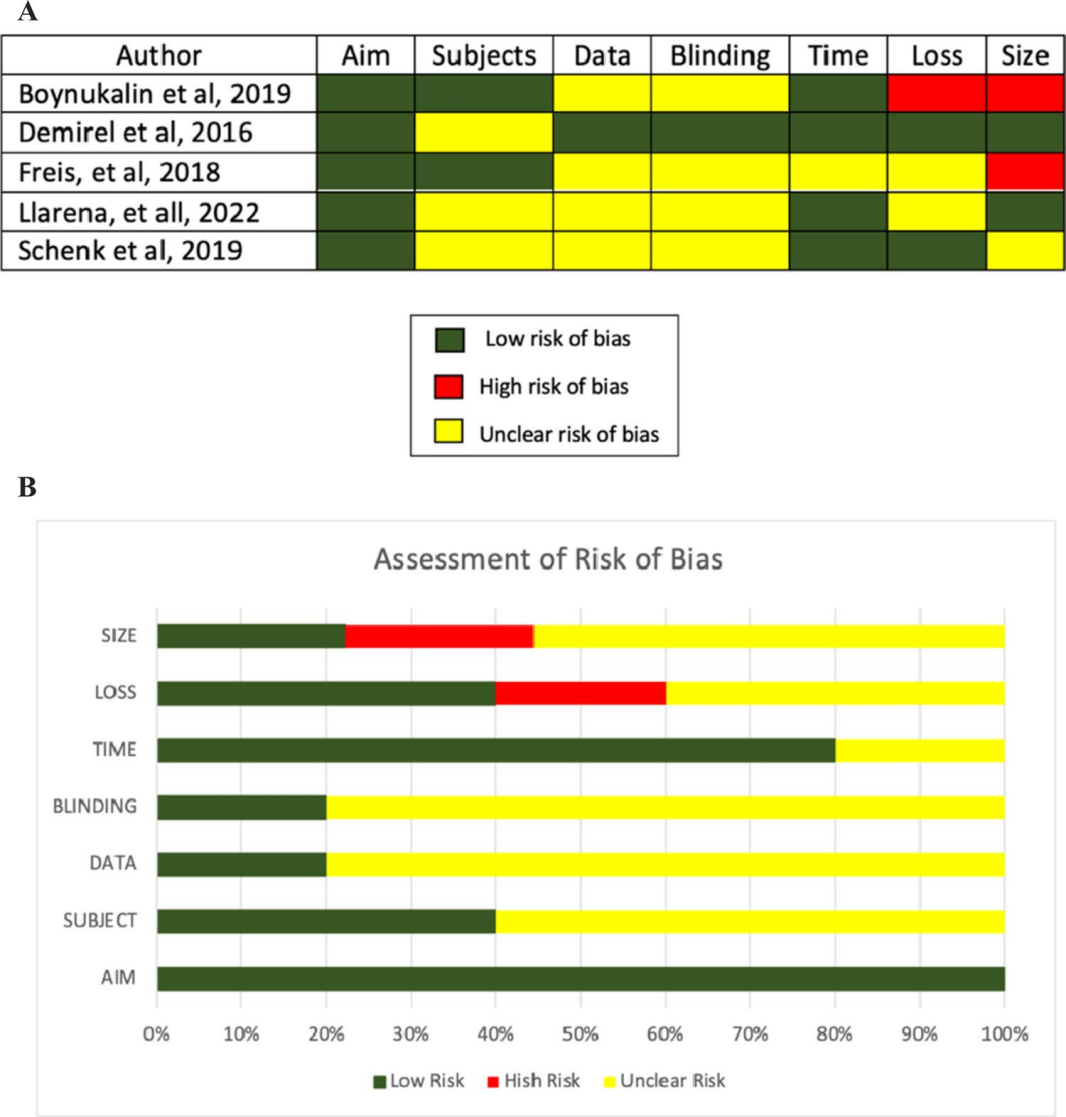
**A, B** Assessment of risk of bias. **A** Summary of risk of bias for each study: green-low risk of bias; red-high-risk of bias; yellow-unclear risk of bias. **B** Risk of bias graph about each risk of bias item presented as percentages in all the included studies

Using the search terms and criteria mentioned above, a total of 159 studies were retrieved and their abstracts evaluated for relevance (Fig. 1). Twenty-five studies were selected in full text, as potentially relevant. Finally, five studies were found to be eligible for inclusion in this paper and for further detailed analysis. The risk of bias assessment of these five studies is shown in Fig. 2.

The five studies included in this review comprised a total of 889 patients: 453 had endometriosis (Endo), diagnosed either surgically or sonographically, and 436 were control consisting of tubal, unexplained or prolonged infertility patients as summarized in Table 1. The results can be categorized as follows:

**Table 1:** Characteristics and morphokinetic results reported in the studies

Author	Type of Study	Inclusion Criteria	Study subjects	Number of Subjects
Boynukalin et al., 2019 [16]	Retrospective observational study	1. Age < 40 2. No male factor infertility 3. No structural or numerical chromosomal errors necessitating preimplantation genetic diagnosis 4. No uterine anomalies or PCOS	53 study patients (diagnosed surgically and sonographically) 30 control patients (tubal infertility)	439 embryos: 1. Study group: 53 patients (264 embryos) 2. Control group: 29 patients (175 embryos)
Demirel et al., 2016 [17]	Observational prospective study	1. Age < 42 2. No previous IVF/ICSI treatment 3. Day 3 FSH < 10 mIU/mL 4. AFC 5 5. At least 2 oocytes from each ovary 6. Planned ICSI and fresh blastocyst transfer on day 5	20 study subjects (diagnosed sonographically): Study group: ovary with endometrioma ≤ 4 cm Control group: normal contralateral ovary	128 embryos from 20 infertility women: 1. Study group: 69 embryos 2. Control group: 59 embryos
Freis, et al., 2018 [18]	Retrospective study	1. Age 18—45 2. No confounding comorbidities (PCOS, pathological spermiograms, structural or numerical genetic abnormalities, preimplantation genetic screening)	72 study patients (diagnosed surgically) 96 control patients (tubal factor, unexplained infertility, or prolonged infertility)	477 embryos: 1. Study group: 72 patients (213 embryos) 2. Control group: 96 patients (264 embryos)
Llarena, et all, 2022 [19]	Retrospective study	1. Ages 18—39 2. No PCOS, recurrent pregnancy loss, unexplained infertility and diminished ovarian reserve	126 study patients (diagnosed surgically or sonographically) 233 control patients (tubal factor, unexplained infertility or prolonged infertility)	3471 embryos: 1. Study group: 126 patients (1078 embryos) 2. Control group: 233 patients (2393 embryos)
Schenk et al., 2019 [20]	Retrospective study	1. BMI 17.5–30 2. Age 26–39 3. No nicotine abuse 4. No endocrine disorders (PCOS, POI, menopause, hypothalamic amenorrhea, CAH) 5. No diabetes mellitus 6. No chronic inflammation 7. No known genetic disorders 8. No severe OATS	86 study patients 77 control patients (unexplained or prolonged infertility)	1148 embryos: 1. Study group: 86 patients (552 embryos) 2. Control group: 77 patients (596 embryos)

### Stimulation protocol, oocyte yield and fertilization

The IVF outcomes for the five studies are shown in Table 2, with those with significant differences highlighted.

**Table 2:** Comparison of stimulation protocol, oocyte yield, mature oocytes and fertilization rates

Study (n = 889 subjects)	Stimulation Protocol	Oocyte yield Endo vs controls	Mature oocytes (MII) Endo vs. controls	Fertilization Endo vs controls
Boynukalin et al., 2019 [16]	GnRH antagonist protocol	**7.50 ± 3.58 vs 8.44 ± 1.21 (*p* < 0.05)**	**5.84 ± 3.24 vs 7.2 ± 0.9 (*p* < 0.01)**	84.5 vs 82.3 (not significant
Demirel et al., 2016 [17]	GnRH antagonist protocol	5.3 ± 3.6 vs 4.3 ± 2.7 (*p* 0.3)	4.2 ± 2.6 vs 3.6 ± 2.4 (*p* 0.5)	82 (69/84) vs 85 (59/69) (*p* 0.7)
Freis, et al., 2018 [18]	Ultralong and long agonist	9.6 ± 5.0 vs 10.0 ± 5.1 (*p* not significant)	–	60.4 ± 23.8 vs 57 ± 23.8 (*p* not significant)
Llarena, et all, 2022 [19]	Antagonist, microdose flare, standard long, agonist –antagonist, and mini-stimulation protocols	**12.6 ± 8 vs 15.2 ± 8.2 (*p* 0.001)**	**8.4 ± 4.8 vs 10.5 ± 5.9 (*p* < 0.001)**	**0.80 [0.67, 0.94] vs 0.78 [0.63, 0.89] (*p* 0.036)**
Schenk et al., 2019 [20]	GnRH antagonist protocol	8.9 [7.8, 10.1] vs 7.9 [7.1, 8.8]	–	–

Comparisons that showed statistically significant differences are shown in bold

### Embryo morphokinetic changes

In total, 5663 embryos from the included studies were extensively studied for embryo morphokinetics under time lapse monitoring

#### First embryo cell cycle (VP and ECC1)

The first embryo cell cycle (ECC1) comprises early morphokinetic parameters which starts from the time the second polar body detaches from the oolemma (tPB2) to the appearance of individual pronuclei (tPNa) until the development of two discrete cells (t2). Out of the five studies, only two evaluated the first embryo cell cycle. In the study of Boynukalin [16], tPB2 and tPNa were noted to be statistically longer in the endometriosis group compared to controls. However, ECC1 was surprisingly significantly shorter in the endometriosis group (Table 3).

**Table 3:** Comparison of outcomes of the first embryo cell cycle

Study	tPB2 Endo vs Controls	tPNa Endo vs Controls	tPNf Endo vs Controls	ECC1 Endo vs Controls	VP Endo vs Controls
Boynukalin et al., 2019 [16]	**6.51 ± 9.07 vs 3.71 ± 1.98 (*p* < 0.01)**	**12.50 ± 7.87 vs 11.13 ± 3.74 (*p* < 0.01)**	25.90 ± 6.31 vs 25.30 ± 7.87 (not significant)	**22.19 ± 8.23 vs 24.56 ± 5.66 (*p* < 0.01)**	13.25 ± 6.23 vs 14.87 ± 7.79 (not significant)
Demirel et al., 2016 [17]	–	–	–	–	–
Freis, et al., 2018 [18]	–	–	–	–	–
Llarena, et all, 2022 [19]	–	–	–	–	–
Schenk et al., 2019 [20]	–	–	25.7 [25.4, 26.0] vs 24.9 [24.6, 25.2]	–	–

Comparisons that showed statistically significant differences are shown in bold

#### Second embryo cell cycle (ECC2)

The second cell cycle (ECC2) is characterized by the division from two discrete cells to four cells. In the study of Llarena [19], embryos of endometriosis patients were significantly slower than controls to complete the t2–4 cell stages. This was consistent with the results of Boynukalin [16], wherein s2 was seen to be significantly longer in the endometriosis group. But all other morphokinetic parameters (t2, t3, t4) and duration events (VP, ECC2a, ECC2) were similar between the endometriosis and control groups. Schenk [20] even observed significantly faster s2 in the endometriosis group (*p* <0.05). Freis [18] also showed that the CS2-4 and DR did not show any significant difference between the two groups (Table 4).

**Table 4:** Comparison of outcomes of second embryo cell cycle

Study	t2 Endo vs Controls	t3 Endo vs Controls	t4 Endo vs Controls	ECC2 Endo vs Controls	S2 Endo vs Controls	CS2-4 Endo vs Controls	DR Endo vs Controls
Boynukalin et al., 2019 [16]	28.64 ± 5.24 vs 28.25 ± 5.40 (not significant)	38.02 ± 6.87 vs 37.67 ± 6.33 (not significant)	41.44 ± 7.35 vs 40.19 ± 6.29 (not significant)	12.87 ± 5.47 vs 12.02 ± 4.73 (not significant)	**3.40 ± 5.31 vs 2.53 ± 4.24 (*p* < 0.01)**	–	–
Demirel et al., 2016 [17]	29.3 vs 28.6 h (*p* 0.2)	–	–	8.9 vs 9.9 h (p 0.5)	3.9 vs 2.6 h (*p* 0.6)	–	–
Freis, et al., 2018 [18]	2.7 (0.7–5.3) vs 2.6 (1.9–8.7) (not significant)	14.2 (6.7–21.6) vs 14.3 (12.1–24.5) (not significant)	15.4 (7.2–37.0) vs 15.0 (12.4–43.7) (not significant)	12.4 (0.0–33.5) vs 12.7 (0 .0–29.4) (not significant)	0.7 (0.0–20.8) vs 0.6 (0.0–10.5) (not significant)	0.1 (0.0–0.8) vs 0.1 (0.0–1.0) (not significant)	0.8 (0.0–8.0) vs 0.8 (0.0–13.1) (not significant)
Llarena, et all, 2022 [19]	**28.1 ± 5.6 vs 27.2 ± 4.7 (*p* < 0.001)**	**38.2 ± 7.1 vs 36.8 ± 5.9 (*p* < 0.001)**	**41.0 ± 8.2 vs 39.5 ± 7.2 (*p* < 0.001)**	**11.3 [9.7, 12.5] vs 11.2 [9.5, 12.2] (*p* 0.003)**	0.67 [0.33, 2.3] vs 0.67 [0.33, 2.2] (p 0.64)	–	–
Schenk et al., 2019 [20]	29.4 [29.0, 29.9] vs 28.5 [28.1, 29.0]	39.1 [38.5, 39.6] vs 37.9 [37.3, 38.5]	41.8 [41.3, 42.4] vs 41.5 [40.9, 42.2]	25.7 [25.4, 26.0] vs 24.9 [24.6, 25.2]	**Significantly lower in the Endo (*p* < 0.05)**	–	–

Comparisons that showed statistically significant differences are shown in bold

#### Third embryo cell cycle (ECC3)

The third embryo cell cycle consists of the time the embryo develops from four cells to eight cells. In the study of Llarena et al. [19], the embryos of endometriosis patients were slower than controls to complete the t5–8 cell stages, occurring 1 to 1.8 h later than controls. CS2–8 was decreased while CS 4–8 was increased in embryos of patients with endometriosis, both indicating inferior embryo development for endometriosis [18]. Nonetheless, the rest of the studies showed no difference in the third embryo cell cycle between endometriosis and control embryos (Table 5). However, Schenk [20] showed that those in the severe endometriosis group, based on rASRM staging, showed significant temporal timing at t9 reaching this stage faster than the minimal endometriosis, moderate endometriosis and control groups (all *p* < 0.01). But the time-gain equalized for all groups, at the end of compaction process (tMor).

**Table 5:** Comparison of outcomes of the third embryo cell cycle

Study	t5 Endo vs Controls	t6 Endo vs Controls	t7 Endo vs Controls	t8 Endo vs Controls	t9 Endo vs Controls	ECC3 Endo vs Controls	s3 Endo vs Controls	CS2-8 Endo vs Controls	CS4-8 Endo vs Controls
Boynukalin et al., 2019 [16]	50.51 ± 9.86 vs 49.76 ± 10.41 (not significant)	55.28 ± 10.14 vs 53.77 ± 9.91 (not significant)	58.11 ± 10.14 vs 58.33 ± 10.28 (not significant)	62.67 ± 11.80 vs 61.45 ± 11.09 (not significant)	71.57 ± 13.37 vs 69.62 ± 11.58 (not significant)	22.56 ± 9.46 vs 22.03 ± 9.30 (not significant)	12.40 ± 9.20 vs 12.59 ± 10.01 (not significant)	–	–
Demirel et al., 2016 [17]	52.3 vs 52.5 h (*p* 0.9)	–	–	–	–	–	–	–	–
Freis, et al., 2018 [18]	28.0 (5.8–55.7) vs 27.9 (19.2–53.0) (not significant)	30.0 (6.8–51.6) vs 29.4 (23.6–64.0) (not significant)	32.8 (8.5–56.0) vs 30.9 (25.2–66.7) (not significant)	34.3 (11.5–68.7) vs 33.0 (25.3–59.9) (not significant)	50.0 (16.8–73.2) vs 50.3 (28.4–74.7) (not significant)	18.0 (3.4–53.6) vs 17.8 (5.5–47.3) (not significant)	5.6 (1.4–36.0) vs 4.5 (0.9–27.5) (not significant)	**0.7 (0.0—0.93) vs 0.8 (0.0–0.94) (*p* < .05)**	**0.4 (0.1–1.0) vs 0.3 (0.1–1.0) (*p* < .05)**
Llarena, et all, 2022 [19]	**51.1 ± 10.5 vs 49.4 ± 9.4 (*p* < 0.001)**	**–**	**59.2 ± 10.9 vs 57.5 ± 10.2 (*p* < 0.001)**	**61.9 ± 12.0 vs 60.0 ± 11.2 (*p* < 0.001)**	**72.2 ± 12.3 vs 70.1 ± 11.8 (*p* < 0.001)**	**13.2 [11.0, 15.7] vs 13.0 [10.9, 15.1] (*p* 0.046)**	–	–	–
Schenk et al., 2019 [20]	52.5 [51.6, 53.3] vs 51.3 [50.4, 52.3]	56.1 [55.3, 57.0] vs 55.4 [54.6, 56.3]	60.0 [59.1, 61.0] vs 59.8 [58.8, 60.7]	63.7 [62.6, 64.9] vs 62.6 [61.5, 63.7]	74.2 [72.7, 75.8] vs 72.8 [71.3, 74.4]	No difference in ECC3	No difference in s3	–	–

Comparisons that showed statistically significant differences are shown in bold

#### Morula formation and blastulation

Only one study discussed the development of the embryos from eight cell to morula and to blastocyst stage. Llarena [19] noted that embryos of endometriosis patients were slower than controls at nearly all late developmental milestones evaluated namely tM, tSB, tB and tEB (Table 6). Significantly fewer endometriosis embryos had optimal morphokinetics for tSB and tEB, even reaching tSB, tB, and tEB 1.5–2 h later compared to controls. When the endometriosis embryos were classified according to disease stage, tSB was longer in rASRM stage 3–4 cohort (mean 104.1±10.7 vs 102.2±9.6, p=0.015) and the timing of blastulation was also delayed by approximately 2 h (mean 109.1±10.3 vs 107.5±10.0, p=0.058) when compared to stage 1–2, but did not reach statistical significance.

**Table 6:** Comparison of morula and blastulation rates

Study	tM Endo vs Controls	tSB Endo vs Controls	tB Endo vs Controls	tEB Endo vs Controls	DC Endo vs Controls	DR Endo vs Controls
Boynukalin et al., 2019 [16]	–	–	–	–	–	–
Demirel et al., 2016 [17]	–	–	–	–	–	–
Freis, et al., 2018 [18]	–	–	–	–	9/72 (12.5%) vs 4/96 (4.2%) (*p* 0.076)	0.8 (0.0–8.0) vs 0.8 (0.0–13.1) (not significant)
Llarena, et all, 2022 [19]	**93.1 ± 11.5 vs 92.1 ± 11.2 (*p* 0.036)**	**103.2 ± 10.3 vs 101.4 ± 10.0 (*p* < 0.001)**	**108.4 ± 10.2 vs 106.4 ± 10.0 (*p* < 0.001)**	**116.5 ± 10.3 vs 114.5 ± 10.1 (*p* < 0.001)**	8.6% vs 9.2% (*p* 0.56)	–
Schenk et al., 2019 [20]	87.4 [86.1, 88.7] vs 86.1 [84.6, 87.6]	–	–	–	17 cases (3.1%) vs 17 cases (2.9%) (not significant)	–

Comparisons that showed statistically significant differences are shown in bold

Three studies evaluated embryo cleavage patterns. In the study of Freis [18] and Schenk [20], direct cleavage was seen higher in the endometriosis embryos, which may indicate lower implantation potential, but was not statistically significant (Table 6). It was only the rate of multinucleation that showed was statistically higher in the endometriosis group (50.0% vs 44.5%, *p*=0.003) [19].

### Embryo quality and blastocyst development

A critical outcome measure in IVF is the rate of blastocyst development and the embryo quality obtained after fertilization. Good-quality embryos were defined by Ciray et al. [13] as having all mononucleated cells, no fragmentation, all cells possessing equal volume and having pearl cytoplasm, while poor-quality embryos are those with at least one cell that is multinucleated, >50% fragmentation, >30% difference in volume between cells and have coarse granularity.

Boynukalin [16] and Llarena [19] showed that the percentage of good quality embryos was significantly lower in the endometriosis group (Table 7). The number of embryos available for transfer likewise appears to be lower in endometriosis group. Among all the retrievals done by Llarena [19], 16 cycles had no embryo available for transfer; 11 (7.4%) of which were in the endometriosis group and only 5 (1.7%) in the control group (OR=4.95; CI: 1.41, 16.84, *p*=0.005). Nonetheless, severity of endometriosis did not appear to influence blastocyst development and quality. Both Llarena [19] and Freis [18] did not report any differences in the rates of embryo wastage and progression to morula, blastocyst, or expanded blastocyst between rASRM stage I–II when compared to stage III–IV.

**Table 7:** Comparison of blastocyst quality, implantation rate and pregnancy rate

Study	Blastocyst Development/Quality Endo vs Controls	Implantation Rate Endo vs Controls	Pregnancy Rate Endo vs Controls
Boynukalin et al., 2019 [16]	**30% vs 54% Day 3 embryos (*p* < 0.01)**	–	41.5% vs 44.8%
Demirel et al., 2016 [17]	15% vs 19% (p 0.3)	–	**50% vs 57% (*p* < 0.05)**
Freis, et al., 2018 [18]	–	–	–
Llarena, et all, 2022 [19]	**59.95% vs 66.2% (*p* < 0.001)**	111/201 (55%) vs 212/385 (55%)	63.6% vs 66.5% (*p* 0.56)
Schenk et al., 2019 [20]	–	29.6% (Min: 33.3%; Mild: 21.2%; Mod: 22.5%, Severe: 14.3%) vs 31.8%	25.2% vs 25.2%

Comparisons that showed statistically significant differences are shown in bold

Boynukalin [16] compared the morphokinetic parameters of good and poor quality embryos in detail. For endometriosis embryos, there were significant differences observed between those rated good versus poor quality in the following parameters: tPB2 t2, t3, t4, VP, ECC1 and ECC2. In the control group, only t5 and ECC2 were found to be significantly different between good and poor quality embryos. ECC2 was the common parameter that showed significant difference between the good and poor quality embryos in both groups. When good- and poor-quality embryos were further subdivided into endometriosis and control groups, tPB2, tPNa, VP and ECC1 were significantly different between good quality endometriosis embryos and good-quality control embryos. On the other hand, morphokinetics between poor-quality endometriosis versus poor-quality control embryos showed no difference.

### Implantation and pregnancy

Pregnancy was uniformly defined as fetal heartbeat 6 weeks after embryo transfer. Four studies noted higher pregnancy rates among the controls when compared to endometriosis patients, but only one reached statistical significance (Table 7) [16, 17, 19, 20]. Implantation rate showed no significant difference between the two groups [19, 20]. Miscarriage rates were also not different between endometriosis patients vs controls (10.7% vs 10.3%, *p*=0.92) [19]. When the different stages of endometriosis was evaluated to determine its impact to pregnancy rates, no difference was noted [19, 20]. Schenk et al. noted that embryos reaching t2 faster were likely to implant and eventually developing a heartbeat (*r* = 0.14, *p* < 0.05, *n* = 250). However, this association was weak and was advised to be interpreted with caution [19].

Only one study did a subgroup analysis of the morphokinetic parameters associated with successful implantation. Llarena et al. showed implantation rate of 55% for both endometriosis and control groups and no difference in pregnancy rate between the two groups [19]. When the morphokinetic parameters of those implanted embryos between endometriosis and control groups were compared, no significant difference were noted. Similar findings were also noted when non-implanted embryos between endometriosis and control groups were compared [19]. However, when the morphokinetics between non-implanted and implanted embryos were compared within each group, significant differences in the parameters were noted. In endometriosis group, t7, tSB and tB occurred significantly longer in non-implanting embryos. For the control group, tM, tSB, tB, tEB and tSC were all substantially delayed in non-implanting embryos (Table 8). Hence, delay in late developmental morphokinetics, primarily compaction, morulation, and blastulation, is associated with failure to implant. Additionally, embryos that had optimal kinetic ranges for s2, t5, tSB and tEB were more likely to implant in the endometriosis and control groups. Llarena et al. also found out that the rates of multinucleation was higher in non-implanting embryos in both endometriosis (43.3% vs 33.3%, *p*=0.019) and control groups (40.5% vs 27.8%, *p*=0.019) [19].

**Table 8:** Summary of intra-group differences between implanting and non-implanting embryos [19]

	Morphokinetic parameter	Non implanting embryo	Implanting embryo	*p* value
Endometriosis group	**t7**	**58.6 ± 8.2**	**55.2 ± 7.1**	**0.003**
	**tSB**	**102.1 ± 9.2**	**97.5 ± 7.1**	** < 0.001**
	**tB**	**106.3 ± 8.6**	**102.5 ± 7.0**	** < 0.001**
	tM	91.7 ± 10.7	87.8 ± 9.0	ns
	tEB	113.9 ± 8.2	110.6 ± 8.1	ns
	tSC	86.0 ± 11.7	81.6 ± 8.8	ns
Control group	**tM**	**91.8 ± 10.7**	**87.7 ± 8.2**	** < 0.001**
	**tSB**	**100.8 ± 7.9**	**97.2 ± 7.3**	** < 0.001**
	**tB**	**105.7 ± 8.1**	**102.3 ± 7.5**	** < 0.001**
	**tEB**	**113.6 ± 8.4**	**109.8 ± 7.1**	** < 0.001**
	**tSC**	**84.7 ± 9.7**	**81.3 ± 9.0**	** < 0.001**

Comparisons that showed statistically significant differences are shown in bold

### Livebirth

Despite differences in embryo morphokinetics between endometriosis and control groups, the implantation, pregnancy, and live birth rates (LBR) have consistently shown no difference between endometriosis and control groups. LBR was only reported in two studies, and both showed no significant difference between the two groups (Table 9). Furthermore, no difference in LBR was found across different stages of endometriosis [20].

**Table 9:** Comparison of live birth rates

Study (*n* = 5,663 embryos)	Livebirth Rate Endo vs Controls
Boynukalin et al., 2019 [16]	–
Demirel et al., 2016 [17]	–
Freis, et al., 2018 [18]	–
Llarena, et all, 2022 [19]	56.1% vs 58.7% (p 0.62)
Schenk et al., 2019 [20]	22.7% vs 24.4% (ns)

## Discussion

Endometriosis is believed to negatively affect oocyte quality leading to poor embryo development and lower pregnancy rates per oocyte retrieved or per embryo transferred [3]. A number of mechanisms have been implicated in this unfavorable effect, including detrimental local inflammatory milieu, increased oxidative stress, impaired steroidogenesis [3], altered immune function of follicular and peritoneal environments [20] and reduction of ovarian tissue function because of endometriomas or past surgery.

Exposure of oocytes to such an environment may induce meiotic abnormalities and chromosomal instability, leading to a reduction in embryo quality [16, 21, 22]. The ability to identify the best embryo, as it develops in vitro using TLM, would make the choice of embryo for transfer more objective and hopefully improve the rate of implantation and LBR. The data on this topic are sparse, as we could only identify five studies on TLM in endometriosis that could be included in this review.

An early meta-analysis by Barnhart et al. showed a lower fertilization rate in endometriosis patients [odds ratio (OR), 0.81; 95% confidence interval (CI), 0.79–0.83, *p* < 0.001)], supporting the negative impact of endometriosis on oocyte quality [23]. However, some studies have shown that the blastocyst formation rate remains unaltered in endometriosis patients. Hamdan et al. showed that women with endometrioma, despite having lower number of oocytes in IVF cycles, have similar reproductive outcomes as those without the disease [24]. Pregnancy rates were reported to be comparable between endometriosis and control patients, as long the overall number of good-quality embryos did not differ significantly between the two groups [4]. Hence, endometriosis appears to primarily affects oocyte quality, but once fertilized and developed into a good-quality blastocyst, pregnancy outcome and rate of live birth appear comparable to those in unaffected women [25].

Several alterations in the morphokinetic parameters of embryos derived from women with endometriosis have been described by some, but not all, of the studies included in this systematic review. Llarena et al. demonstrated that embryos obtained in endometriosis patients had significant delays in both early and late developmental events [19]. Boynukalin et al. reported substantial delay in in the early post-fertilization and first cleavage time points the endometriosis group compared to the control group [16]. Freis et al. showed poorer relative morphokinetic parameters (CS2–8 and CS4–8) in the endometriosis group [18].

The etiology of these endometriosis-associated alterations in embryo morphology are likely multi-factorial. Good-quality cytoplasmic and nuclear components of oocytes, like adequate mitochondria and functional cytoskeleton, are necessary for embryos to attain optimal early cleavage times [26]. These cellular components may be insufficient in the oocytes of endometriosis patients. Alterations in hormonal levels and increased exposure to cytokines, such as TNF-a and ROS, are other factors that may negatively influence early embryo morphokinetic events of patients with endometriosis [22]. Significant delays in polar body extrusion and in early morphokinetic events in embryos from endometriosis patients may make them more prone to chromosomal errors, and thus result in embryos with lower implantation potential [22, 27]. Moreover, endometriosis embryos were less likely to develop to morula, blastocyst, and expanded blastocyst stages when compared to controls [19].

However, unexpected findings in the morphokinetic parameters of endometriosis embryos were also seen in some studies. Demirel et al. showed that embryos derived from oocytes collected from endometrioma-containing ovary have comparable early embryo morphokinetics when compared to those obtained from the contralateral normal ovary [16]. It is important to note that the authors evaluated intra-patient differences and did not include patients without endometriosis. The study by Schenk et al. noted that the synchronicity of the two blastomere divisions in s2 was found to be faster in the endometriosis embryo group when compared to the control group [19]. The duration of the first cell cycle (ECC1) was also shorter in embryos of endometriosis patients in the study of Boynukalin et al. [16]. These unexpected embryonic parameters in endometriosis patients could be due to cellular rearrangement processes and DNA repair mechanisms triggered by oxidative stress that may hasten of early cell division in embryos affected with endometriosis [28]. It is important to note that these were relatively small studies which analyzed 264 and 553 embryos, respectively, and might not have had adequate power to show differences between groups, in contrast to the larger study of Llarena which analyzed 1078 embryos [19].

The novelty of this systematic review is that we summarize and discuss specific TLM parameters from the cell cycle in embryos from women with endometriosis compared to those without disease. Several of the TLM parameters show promise in identifying a higher quality embryo with better implantation potential in women with endometriosis. Based on this review, these include:

1. Optimal kinetic range for ECC2 (> 5 and ≤ 11.9 h) and t5 (45–57 h) [19].
2. Timely start of blastulation (< 96.2 h) and timing of expanded blastocyst (≤ 116 h) [19]. Previous reports had shown that delayed initiation of blastulation correlated with poor blastocyst quality, decreased implantation and aneuploidy [29–31].
3. Direct cleavage (DC) or embryo cleavage from two to three cells (t3-t2) in less than 5 h [18, 32]. DC was noted to be more common in patients with endometriosis than in controls (12.5% vs 4.2%), though this did not reach statistical significance (*p* < 0.076), likely due to inadequate sample size [18].
4. Higher rates of multinucleation in embryos were associated with lower implantation [19].

Interestingly, the adverse impact of endometriosis on embryonic development, as seen through aberrations in morphokinetic parameters, appears to be independent of the stage of disease [18, 19]. Additional studies evaluating whether medical or surgical treatment of endometriosis may improve previously observed alterations in embryo morphokinetic parameters need to be performed.

Despite these reported differences in morphokinetic parameters, clinical pregnancy and LBR did not significantly differ between the endometriosis and control groups [8, 10, 19, 20]. These results are consistent with the meta-analysis by Hamdan et al. that showed similar clinical pregnancy and LBR between endometriosis and unaffected patients, despite higher rate of cycle cancellation and a lower number of retrieved oocytes among endometriosis patients [24]. This implies that once a good quality embryo is available for transfer, regardless if the patients has endometriosis or not, implantation and pregnancy rates will still be comparable. Women with endometriosis had a higher rate of failure to produce an embryo available for transfer, which could be attributed to lower ovarian reserve, higher cycle cancellation and lower number of oocytes retrieved from these patients.

The routine use of TLM as standard practice for IVF has recently been questioned. In a meta-analysis of six randomized controlled trials (RCTs) by Magdi et al., embryo selection using TLM was associated with higher live birth rates and lower early pregnancy loss than conventional embryo selection [33]. However, a very recent meta-analysis which included 14 RCTs showed that embryo selection using TLM did not improve live birth rate, ongoing pregnancy rates, or implantation rates when compared with conventional morphological selection [34]. Thus, although the current evidence for implementing TLM as a standard procedure in IVF remains weak, its use in specific populations, especially in women with endometriosis, deserves further study.

Several limitations of this systematic review need to be acknowledged. The studies included were all retrospective and observational in design. None of the studies included reported using morphokinetic parameters as basis for embryo selection. It remains unclear whether choosing an embryo for transfer based on specific morphokinetic parameters increases the rate of implantation, pregnancy and livebirth. Thus, the generalizability of these findings needs to be confirmed in prospective studies. Another limitation is that endometriosis severity was assessed using the rASRM staging system. Although well accepted and relatively easy to apply, this staging system may not accurately depict deep infiltrating lesions in the pelvis nor the presence of adenomyosis [33–35]. Both of these factors have been shown to affect implantation and pregnancy outcome, independent of embryo quality.

## Conclusion

Endometriosis has been known to cause damaging effect in oocyte quality and embryo development among patients undergoing IVF. Time lapsed monitoring has allowed us to continually monitor embryo development hence providing us a unique insight into the subtle derangements, possibly brought about by endometriosis, on embryo morphokinetics. Based on the included studies in this review, optimal kinetic parameters in t3-t2, t5, ECC2 and blastulation among embryos with endometriosis were noted to have higher embryo quality and higher implantation rates. However, clinical pregnancy and livebirth rates did not differ between endometriosis and control subjects, despite differences in embryo morphokinetics, regardless of disease severity. The studies included in this review have taken the first step to evaluating the utility of embryo morphokinetics in the selection of good quality embryos in endometriosis. Additionally, the differences they identified in morphokinetic parameters improve our understanding of how endometriosis affects different developmental time points in embryo development. The goal of future prospective studies should be to apply the parameters identified for choosing the embryo with the best potential for implantation, pregnancy, and live birth.

### Supplementary Information

Below is the link to the electronic supplementary material.

Supplementary file1 (DOCX 425 KB)
