# Supplementary material for: Does endometriosis inflict harm on embryos? A systematic review of embryo morphokinetics analysed by time lapse monitoring in women with endometriosis
Source: Arch Gynecol Obstet. 2023 Dec 8;309(4):1191–203. doi: 10.1007/s00404-023-07293-1 (PMC10894102; doi:10.1007/s00404-023-07293-1)
Supplement: Supplementary file 1 — Supplementary file1 (DOCX 425 KB) [file 404_2023_7293_MOESM1_ESM.docx]

**Supplemental Figure**  Definitions for the dynamic monitoring of human preimplantation embryo development

*As previously published in Human Reproduction, Vol number 29, Ciray HN, Campbell A, Agerholm IE, Aguilar J, Chamayou S, Esbert M, et al., Proposed guidelines on the nomenclature and annotation of dynamic human embryo monitoring by a time-lapse user group, Page No 2650–2660, Copyright by Oxford University Press on behalf of the European Society of Human Reproduction and Embryology, 2014, permission from publisher received)*

| **Time** | **Definition of Events** |
| --- | --- |
| ECC1 | Duration of 1st embryo cell cycle: t2-tPB2 |
| VP | PN duration: tPNf-tPNa |
| ECC2 | Duration of second embryo cell cycle: t4-t2 |
| S2 | Synchronization of cell divisions: t4-t3 |
| CS2-4 | Cleavage synchronicity from 2- to 4-cell stage |
| DR | DNA replication time ratio |
| ECC3 | Duration of 3^rd^ embryo cell cycle: t8-t4 |
| s3 | Synchronization of cleavage pattern: t8-t5 |
| CS2-8 | Cleavage synchronicity from 2- to 8-cell stage |
| CS4-8 | Cleavage synchronicity from 4- to 8-cell stage |

Legend:

| **Time** | **Definition of Events** |
| --- | --- |
| tPB2 | Detachment of 2^nd^ polar body from oolemma |
| tPN | Fertilization is confirmed |
| tPNa | Appearance of individual pronuclei |
| tPNf | Time of pronuclei disappearance |
| t2-t9 | Two to nine discrete cells |
| tM | Morulation |
| tSB | Start of blastulation |
| tSB | Initiation of blastulation |
| tEB | Expanded blastocyst |
| DC | Direct cleavage |
| DR | DNA replication time ratio |
